# Chest radiographs of cardiac devices (Part 2): Ventricular assist devices

**DOI:** 10.4102/sajr.v23i1.1732

**Published:** 2019-07-31

**Authors:** Rishi P. Mathew, Timothy Alexander, Vimal Patel, Gavin Low

**Affiliations:** 1 Department of Radiology and Diagnostic Imaging, Faculty of Medicine and Dentistry, University of Alberta, Edmonton, Canada

**Keywords:** Left ventricular assist device, right ventricular assist device, intra-aortic balloon pump, extracorporeal membrane oxygenation, berlin heart, heartmate II, heartware LVAD, heartmate III

## Abstract

Heart failure is considered a worldwide pandemic affecting 26 million people globally. Patients who are unfit or waiting for cardiac transplantation may benefit from alternate mechanical support therapies using ventricular assist devices. It is not uncommon for radiologists, especially those working in institutions with a high volume of cardiac transplantations, to be presented with radiographs containing these devices. The role of the radiologist is not only to accurately identify these devices, but also to evaluate for any complications.

## Introduction

Heart failure (HF) is considered a worldwide pandemic affecting 26 million people globally. Heart failure is characterised by the decreased ability of the heart to pump and/or fill with blood.^1^ In the United Kingdom, approximately 500 000 people are affected,^2^ while in the United States it is approximately 5.1 million. The gold standard treatment for HF is cardiac transplantation, but only a select few go on to receive this treatment, either because of donor shortage or being unfit for surgery. Many patients may benefit from alternate mechanical support therapies using ventricular assist devices (VADs). Rapid advances in medical technologies over the last few decades have produced a myriad of new devices and improvements in existing ones. Chest radiographs (CXRs) are typically obtained in these patients, and it is not uncommon for the radiologist to be presented with a CXR containing a cardiac device. The role of the radiologist involves identifying the device on the CXR, as well as careful assessment for accurate placement and excluding complications. The intention of this article is to provide insight into the various VADs found in clinical practice, identifying the most commonly used types, assessing their placement and highlighting complications on CXRs.

## Ventricular assist devices

Ventricular assist devices are used in patients with ventricular dysfunction – right, left or biventricular – and can be temporary or permanent, based on the clinical indication. Temporary VADs are used as a bridge to myocardial recovery or during cardiac transplantation. Permanent VADs may be used for the same reason but are typically selected in patients who require long-term treatment for myocardial dysfunction and who are not fit for cardiac transplantation.

### Temporary ventricular assist devices

#### Intra-aortic balloon pump

**FIGURE 1:**
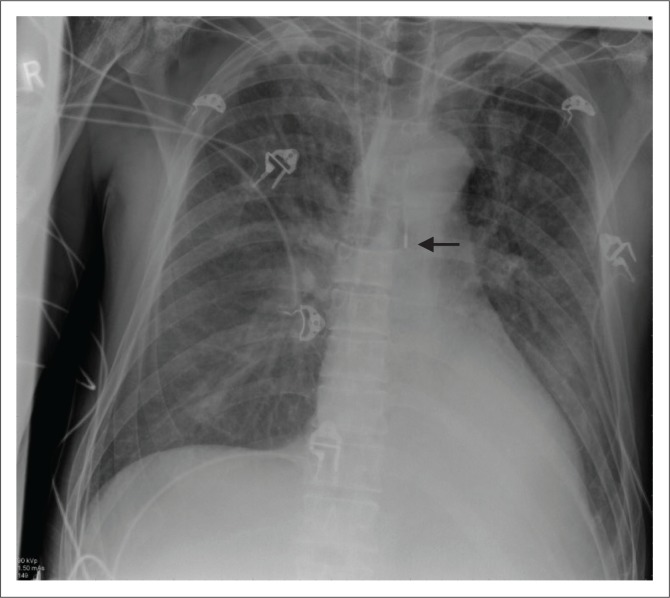
Chest radiograph showing an intra-aortic balloon pump placed in a 53-year-old male with acute post-infarction ventricular septal rupture and cardiogenic shock. Note that the radiopaque tip of the intra-aortic balloon pump (arrow) is at or below the level of the carina and needs to be advanced by 2 cm.

An intra-aortic balloon pump (IABP) is a polyethylene balloon that spans the entire length of the thoracic aorta, placed percutaneously via femoral artery access. The IABP comes in different lengths with IABP selection determined based on the patient’s height. The IABP inflates during diastole leading to an increase in blood flow to the coronary arteries, great vessels and renal arteries. Immediately prior to systole, it deflates producing a vacuum effect leading to forward blood flow to the aorta and its branches. Although the IABP is predominately radiolucent on a CXR, it has radiopaque tips proximally and distally. On the CXR, the cephalad radiopaque tip should be 2 cm above the carina (Figure 1). An alternate landmark would be the aorto-pulmonary window. Placing the IABP too caudally may occlude the celiac, superior mesenteric or renal arteries, while placing it too high may occlude the brachiocephalic, subclavian or carotid arteries. Complications that can occur with IABP include vascular (e.g. limb ischaemia, renal insufficiency, mesenteric ischaemia and aortic dissection) and non-vascular (e.g. catheter-related [perforation, tear and incorrect positioning] infection and neurological sequelae).^3,4,5^

#### Extracorporeal membrane oxygenation cannula

Extracorporeal membrane oxygenation (ECMO) is increasingly being employed in adult patients who fail to wean after cardiopulmonary bypass post-cardiac surgery, as well as in patients with severe respiratory failure. The intention of ECMO is to provide oxygenated blood while extracting carbon dioxide. The two main types of ECMO devices are veno-arterial (VA) ECMO and veno-venous (VV) ECMO. Both allow respiratory support, but only VA ECMO allows haemodynamic support, and hence only the latter is used for HF. In VA ECMO, deoxygenated blood is siphoned off from a vein and oxygenated blood is returned to an arterial vessel (aorta for central VA ECMO and proximal femoral, axillary or subclavian artery for peripheral VA ECMO), while in VV ECMO, the oxygenated blood is returned to a systemic vein or the right atrium. Following appropriate placement, the cannulas are sutured to the skin to prevent displacement and malposition. Radiographs of the chest and abdomen are performed to ensure correct cannula positioning.

**FIGURE 2:**
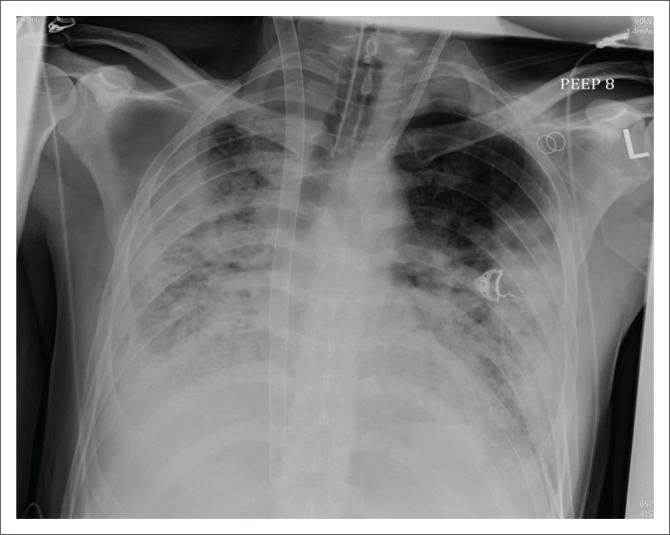
Chest radiograph showing a veno-venous extracorporeal membrane oxygenation with the Avalon catheter inserted from the right internal jugular vein directly into the superior vena cava, right atrium and inferior vena cava in a 38-year-old male with idiopathic pulmonary fibrosis.

**FIGURE 3:**
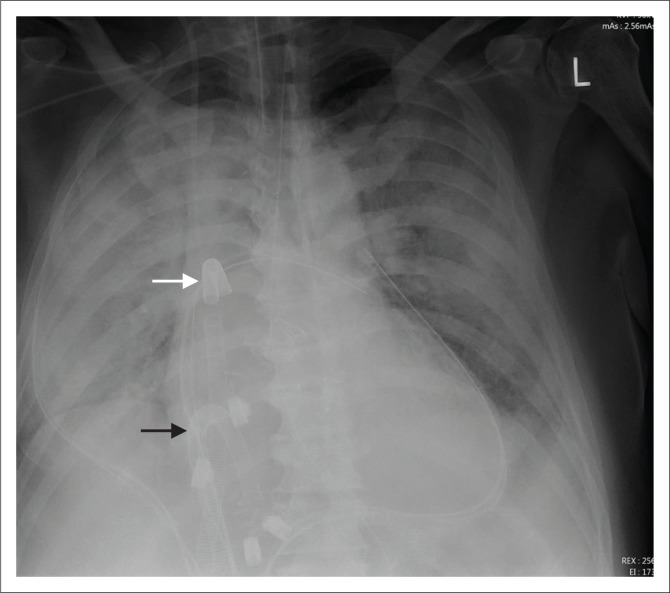
Chest radiograph showing a veno-venous extracorporeal membrane oxygenation with the cannulae in the superior vena cava (white arrow) and right atrium (black arrow) in a 66-year-old male with congestive heart failure (CHF) and pulmonary oedema.

**FIGURE 4:**
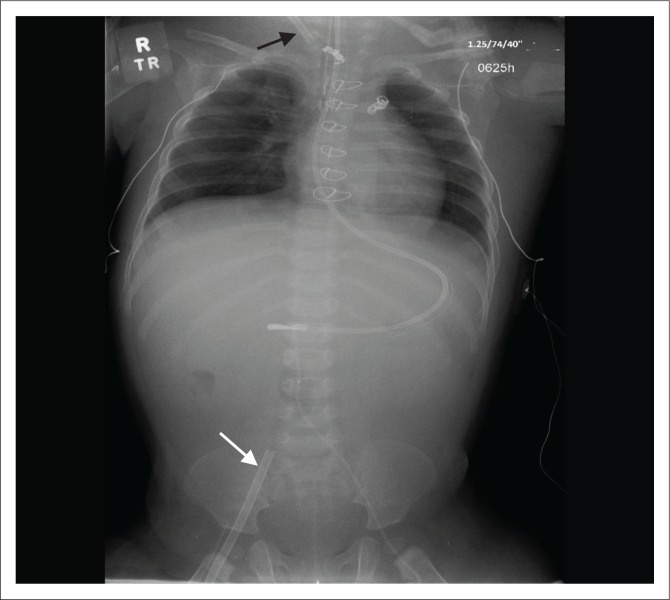
Chest radiograph showing a veno-arterial extracorporeal membrane oxygenation placed in a 1-year-old male child with respiratory failure, with the arterial cannula in the right internal carotid artery (black arrow) and the venous cannula in the right femoral vein (white arrow).

In VV ECMO, the femoral cannula tip should be at the junction of the inferior vena cava (IVC) and the right atrium (RA), and the internal jugular vein (IJV) cannula tip at the junction of the superior vena cava (SVC) and the RA. The VV ECMO dual lumen cannula (e.g. Avalon Elite) is inserted through the right IJV into the RA, with the tip in the IVC (Figures 2 and 3). Cannulation sites for VA ECMO cannulas depend on the device configuration, with femoral-femoral being the commonest^3,6^ (Figure 4). A change in the position of the cannula with reference to an adjacent bony landmark on subsequent radiographs should prompt the radiologist to check for ECMO malfunction. Misplacement of the ECMO cannula can lead to vessel obstruction or occlusion (e.g. SVC obstruction). Other complications that have been reported include gas emboli, thrombosis of veins or arteries, cerebral ischaemia or stroke and haemorrhage because of anticoagulation.^6^ A summary of the various ECMO configurations and their indications are elaborated in Table 1.

**TABLE 1:** Various extracorporeal membrane oxygenation configurations and their indications.

Cannula tip position	Drainage cannula position	Return cannula position	ECMO cannulation indications
**VA ECMO**			
Central	Right atrium	Aorta	For cardiopulmonary support, for example, cardiac failure or failure to wean from cardiopulmonary bypass after cardiac surgery.
Peripheral	Distal IVC or SVC, before the cavoatrial junction	Proximal femoral artery, axillary artery, subclavian artery	-
**VV ECMO**			
Femoro-femoral	Distal IVC, at the level of the diaphragm	Right atrium via the same or opposite iliofemoral vein	Reversible respiratory failure with normal heart function
Femoro-atrial	Distal IVC, at the level of the diaphragm	Distal SVC/right atrium via the SVC	-
Dual lumen, single cannula	IVC, below the diaphragm	Right atrium via the SVC	-

VA, veno-arterial; VV, veno-venous; ECMO, extracorporeal membrane oxygenation; IVC, inferior vena cava; SVC, superior vena cava.

#### Long-term ventricular assist devices

The main indication for these devices is a bridge to cardiac transplantation. In Australia, in 2013, nearly 40% of the patients who underwent heart transplant were bridged to transplantation with a long-term VAD. A list of the various indications and contraindications for left ventricular assist devices (LVADs) are summarised in Table 2.^7^

**TABLE 2:** Indications and contraindications for left ventricular assist devices.

LVAD	LVAD indications and contraindications
Indications	*Bridge to transplantation:* patients awaiting cardiac transplantation *Bridge to candidacy: LVAD* implanted to clarify or improve an aspect of patients’ candidacy prior to transplantation *Bridge to recovery:* rarely indicated in <5% *Destination therapy:* patients not considered for heart transplantation and for long-term strategy
Contraindications	Irreversible end organ failure (hepatic/renal) Metastatic cancer Cerebral damage/neurological deficit/unresolved psychosocial issues Coagulation disorders Mechanical heart valves (may replace with bioprosthetic valves) Severe aortic valve regurgitation Bleeding disorders

LVAD, left ventricular assist device.

The components of a left ventricular assist device in general include the inflow cannula, the impeller (pump), outflow cannula, the percutaneous driveline connected to a power source and the external system controller. The impeller mechanism enables forward blood flow.

**FIGURE 5:**
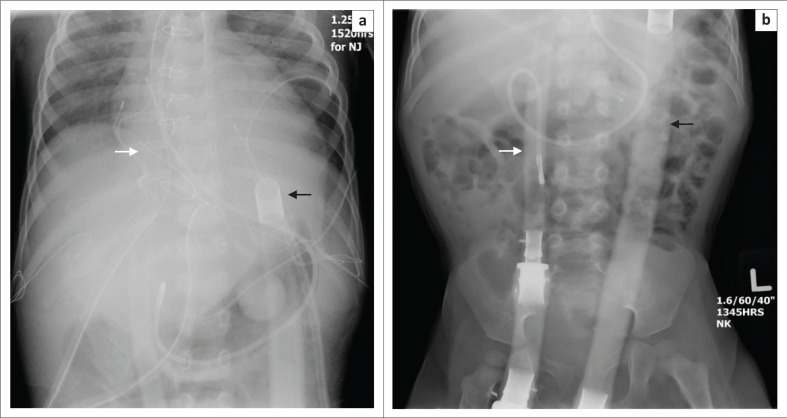
Chest (a) and abdominal (b) radiographs showing a Berlin Heart left ventricular assist device with its inflow (black arrow) and outflow (white arrow) cannulae implanted in the left ventricle and aorta, respectively, in a 2.5-year-old male child with severe left ventricular dysfunction following an episode of myocarditis.

**FIGURE 6:**
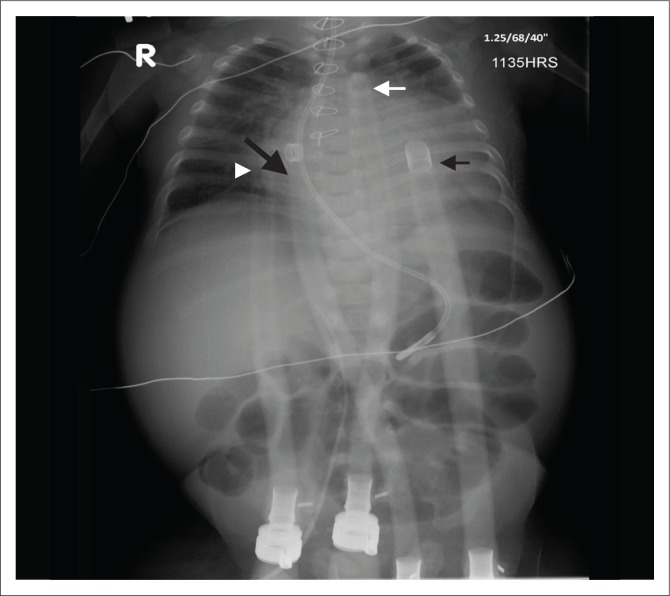
Chest radiograph showing the biventricular assist device (BIVAD) Berlin Heart placed in a 2-week-old infant with severe left ventricular dysfunction and right ventricular hypoplasia. Note the inflow (right black arrow) and outflow cannulas of the left ventricular assist device in the left ventricle and aorta, respectively, while the inflow (arrow head) and outflow (angled arrow) cannulas of the right ventricular assist device are in the right atrium and pulmonary artery, respectively.

*First-generation LVADs*, also known as volume displacement devices, used a volume displacement device with valves to pump out blood. Because of their poor long-term durability, thrombus formation and increased risk for infections, they have fallen out of favour clinically. Currently, the only adult first-generation VAD still available, albeit rarely used, is the Thoratec VAD. It is a pneumatically driven pump and available as either paracorporeal (Thoratec PVAD) or intracorporeal (Thoratec ventricular assist device [IVAD]) models, both capable of providing univentricular (left or right) and biventricular support. The Thoratec PVAD is implanted on the anterior abdominal wall, while the IVAD is implanted in a pre- or intraperitoneal position.^8^ The Berlin Heart EXCOR^®^ Ventricular Assist Device (Berlin Heart, Berlin, Germany), a first-generation VAD, is the most commonly used LVAD worldwide in paediatric patients with end-stage HF as a bridge to heart transplantation.^9^ It consists of a pneumatically driven paracorporeal translucent pump. The device cannulae are made of silicon rubber and coated externally with Dacron velour at the site where it exits the upper abdominal wall to encourage granulation tissue formation and reduce the risk of infection. The device can provide both univentricular and biventricular support (Figures 5 and 6).^10^

*Second-generation LVADs* are currently the most frequently used. Worldwide, the HeartMate II^®^ (HMII) is the most commonly used second-generation LVAD device. The inflow and outflow cannula placements for this device are like the first-generation LVADs. The HMII can be differentiated from other LVADs as they are placed within the subdiaphragmatic preperitoneal pocket and are therefore visualised below the left hemidiaphragm on a CXR. In addition, the impeller housing unit has a characteristic ‘bell-shaped’ appearance, with its percutaneous single driveline supplying power to the pump exiting from the right upper quadrant of the abdomen.^11^ During surgical placement, the inflow cannula is oriented posteriorly towards the centre of the left ventricle (LV) cavity and connected to the pump body at roughly a 30° angle, while the outflow cannula is oriented upward to direct blood flow above the diaphragm. Malpositioned HMII pumps contribute to nearly 25% of pump thrombosis. Other factors that may contribute to pump thrombosis or failure are inflow cannula malposition and outflow cannula kink or compression.^12^

**FIGURE 7:**
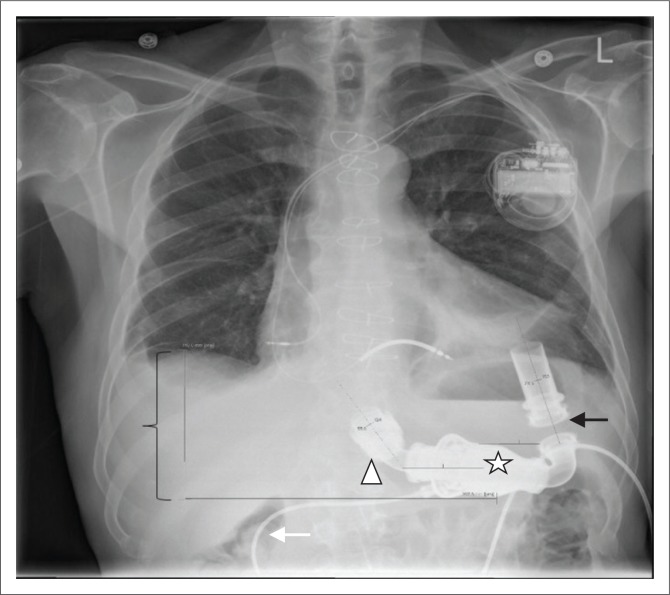
An accurately positioned HMII left ventricular assist device in a 67-year-old male with ischaemic cardiomyopathy. Note that the inflow cannula (black arrow) angle is 72° and the left ventricular assist device is placed at pump pocket depth (left brace) of 11 cm. Note the other parts of the HMII left ventricular assist device – the bell-shaped impeller unit (star), the outflow cannula (arrow head) and the driveline (white arrow).

**FIGURE 8:**
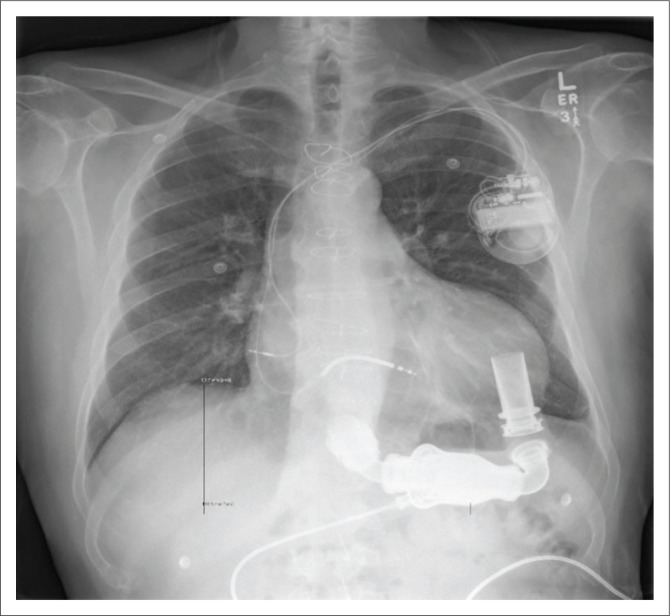
Follow-up chest radiograph after 1 year showed that the same HMII device had migrated cranially by 2 cm.

An acceptable CXR, to reduce the errors from patient rotation on inflow angle measurements, would be one where the sternotomy sutures are seen overlying the patient’s spine or with rotations < 2 cm. The various HMII measurements that may be assessed on an AP CXR include: (1) the pump pocket depth (PPD), (2) the outflow cannula angle and (3) the inflow cannula angle. The outflow cannula is fixed to the body of the HMII with a known angle (generally >100°), and any deviation from the known angle is indicative of a CXR set-up error and rotation. It has been shown that the inflow cannula angle measurements should remain constant on serial imaging. An ideal inflow cannula angle should be >55°–65° to reduce the risk of pump thrombosis (Figure 7). Also, the inflow cannula should point to the central aspect of the left ventricle on a CXR. A deeper PPD (12 cm–14 cm) is believed to help protect against pump thrombosis. However, PPD measurements are not always precise, as the diaphragm is not a fixed structure, and the bottom of the pump on the CXR may not be the actual nadir of the pocket. Also, with time the PPD decreases by 2 cm–2.5 cm because of cranial migration of the pump, even with fixation (Figure 8).^12,13^ A sudden onset of pulmonary oedema could suggest LVAD failure. Other complications that may be seen on CXR include pneumothorax, pneumomediastinum, pericardial tamponade and infections. Documented sites of infection include the superficial driveline, pump pocket, the mediastinum and the internal parts of the pump for which CT is more superior than CXR for evaluation. Occasionally, because of device malfunction, the body of the HMII may be replaced without changing the outflow graft, and as a result of variations between the new and old designs, complications such as outflow bend relief detachments and kinking may be seen.^5,14^

**FIGURE 9:**
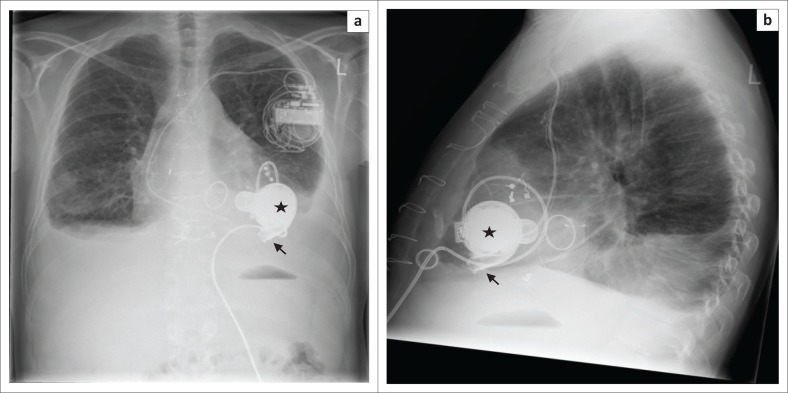
Frontal (a) and lateral (b) chest radiographs showing a HeartWare left ventricular assist device in a 41-year-old male with familial dilated cardiomyopathy. Note that its outflow cannula (arrow) is without angulation and it has a circular impeller unit (star).

**FIGURE 10:**
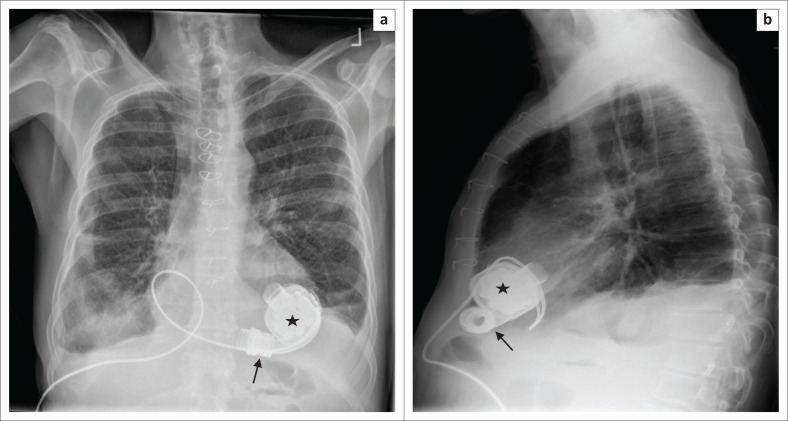
Frontal (a) and lateral (b) chest radiographs showing a HeartMate III device implanted in a 58-year-old male with severe left ventricular dysfunction. Note that the HMIII device has an angled outflow cannula (arrow) and a circular impeller unit (star).

*Third-generation LVADs* use magnetic levitation technology (MAGLEV) enabling rotation without friction or wear, and the pump mechanism produces centrifugal flow. In addition, because of their smaller size, they are implanted in the pericardial space. On CXRs, the impeller units of the HeartWare LVAD (HVAD) and the HeartMate III (HMIII) can be differentiated from HMII, as the former have a circular appearance with a centrally located inflow cannula. The differentiating feature between the HVAD and HMIII devices are that the radiopaque portion of the HMIII’s outflow cannula is angled, while in the HVAD it has a more linear orientation (Figures 9 and 10).^3,14,15^ Currently, the most commonly used third-generation LVADs is the HVAD.^16^ Although echocardiography and CT are the main modalities for detailed evaluation of LVADs, as in the second-generation LVADs, CXRs can also be used to assess for complications related to third-generation LVAD placement. As none of the LVADs from any generation are MR safe, MRI is contraindicated and has no role in their evaluation.^17^ Abdominal radiographs are useful for assessing LVAD driveline damage in the form of fraying or kinking, as damages occurring to the percutaneous portion of the driveline are not evident on visual inspection.^18^

**FIGURE 11:**
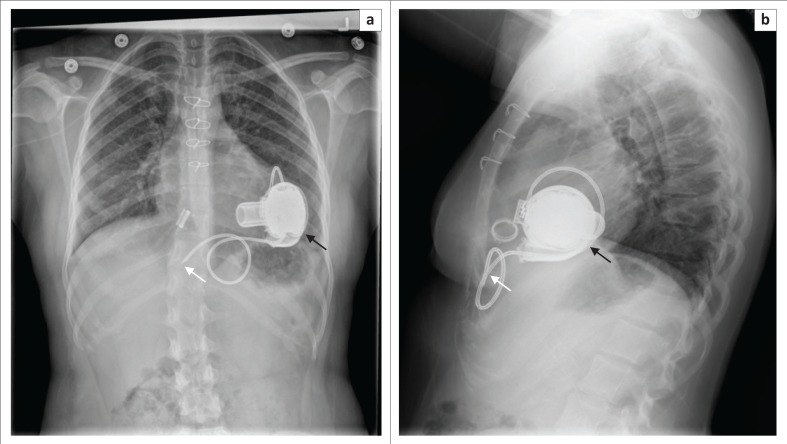
Frontal (a) and lateral (b) chest radiographs showing a decommissioned HeartWare left ventricular assist device (thick arrow) with an intentionally severed driveline (arrow) in a 33-year-old lady with a history of left coronary dissection.

Myocardial recovery is a known phenomenon in patients with LVADs. Although an LVAD explant would offer a better quality of life and freedom for these patients, many centres have hesitancy to perform these procedures as it can be aggressive and there are no established criteria for the device explant. In addition, re-inserting an LVAD in a patient with a previous explant following a relapse of HF can be a challenging task. A minimally invasive LVAD decommission procedure that some follow, includes leaving the device *in situ*, severing the driveline and ligating the outflow graft through a small thoracotomy. This essentially leaves a natural opening for future LVAD reimplantation, should it be needed (Figure 11a and b).^19^

Neurological complications are one of the most devastating and common complications in patients with LVADs. In those patients with a risk for cerebrovascular disease, a pre-operative carotid ultrasound is often done as a standard evaluation. In those patients with neurological symptoms, a post-operative carotid ultrasound is performed. Left ventricular assist devices can alter the waveforms on carotid Doppler and findings include a parvus tardus waveform with a low peak systolic velocity and continuous diastolic flow.^20^

A summary of the classification of various VADs and the characteristics of the various permanent LVADs are outlined in Box 1.

BOX 1 Classification of ventricular assist devices and summary of the permanent ventricular assist devices.

**Classification of VADs:**

Temporary VADs:

1. Percutaneously inserted temporary VADs:
  - IABP, ECMO, Impella devices and TandemHeart
2. Surgically implanted temporary VADs:
  - BVS 5000 and AB5000 (both by Abiomed) and CentriMag (by Thoratec)

Permanent VADs:

1. *First-generation VADs:*
  - Paracorporeal pumps:
    - Thoratec’s paracorporeal ventricular assist devices (PVAD; Thoratec Inc., Pleasanton, CA, USA)
    - Berlin Heart EXCOR^®^ (Berlin Heart AG, Berlin, Germany)
  - Implantable pumps:
    - HeartMate XVE (Thoratec Inc.)
    - Novacor (World Heart Corp., Oakland, CA).
2. *Second-generation VADs*:
    - HeartMate II (HMII; Thoratec Corp., Pleasanton, CA)
    - Incor Berlin Heart (Berlin Heart AG, Berlin, Germany)
    - Jarvik 2000 FlowMaker (Jarvik Heart, Inc., New York, NY)
3. *Third-generation VADs*:
    - HeartWare HVAD System (HeartWare, Framingham, MA)
    - HeartMate III (Thoratec/ St Jude Medical)

**Summary of the various permanent VADs:**

1. First-generation VADs:
  - Pump mechanism: pulsatile flow (volume displacement pumps)
  - Disadvantages: increased risk of infection, large size, poor durability.
  - No longer used except for Thoratec and Berlin Heart EXCOR^®^.
  - Implantation site:
    - Thoratec PVAD: paracorporeal
    - Thoratec IVAD: preperitoneal
    - Berlin Heart EXCOR^®^: preperitoneal
    - HeartMate XVE: preperitoneal or intraperitoneal
    - Novacor: preperitoneal
2. Second-generation VADs:
  - Pump mechanism: continuous (axial flow)
  - Most commonly used VAD. Fewer moving parts when compared to the previous generation (the rotor is the only moving part) and therefore more durable
  - Implantation site: HMII is implanted below the subdiaphragmatic preperitoneal space. The Jarvik and Incor Berlin Heart are implanted in the pericardial space
3. Third-generation VADs:
  - Pump mechanism: continuous (centrifugal) flow
  - Uses magnetic levitation technology (MAGLEV), thereby enabling rotation without friction or wear, more durable, compact size, no pump pocket, simpler surgical implantation
  - Implantation site: pericardial space

VAD, ventricular assist devices; IABP, intra-aortic balloon pump; ECMO, extracorporeal membrane oxygenation.

## Ethical consideration

All ethical considerations have been taken into account. No patient identity or patient information has been revealed.

## Conclusion

Ventricular assist devices play a crucial role in the management of patients with HF. With recent advances, newer devices have become smaller and more durable. The role of the radiologist is to identify the salient medical device on the radiograph, to evaluate for appropriate device placement and to exclude device-related complications.
